# Quality of Pepper Seed By-Products: A Review

**DOI:** 10.3390/foods11050748

**Published:** 2022-03-03

**Authors:** Tanja Cvetković, Jasmina Ranilović, Stela Jokić

**Affiliations:** 1 Research and Development, Podravka Ltd., Ante Starčevića 32, 48000 Koprivnica, Croatia; tanja.cvetkovic@podravka.hr (T.C.); jasmina.ranilovic@podravka.hr (J.R.); 2 Faculty of Food Technology Osijek, Josip Juraj Strossmayer University of Osijek, Franje Kuhača 18, 31000 Osijek, Croatia

**Keywords:** pepper seeds, oil, by-products, *Capsicum annuum*

## Abstract

Peppers are grown all around the world, usually for fresh consumption, as well as for the industrial production of different products. Pepper (*Capsicum annuum* L.) seeds are mostly considered a by-product. Recent investigations have shown that pepper seeds have the potential to be a valuable source of edible oil and fiber-rich flour and protein after processing. Pepper seed oil is a high-quality edible oil according to quality analysis (nutritional, chemical, sensory and antioxidant characteristics) and is suitable as an ingredient for use in the food and nonfood industries (pharmaceutical, chemical, cosmetic industries). The literature review presented in this paper revealed the high quality of two pepper seed by-products (pepper seed oil and pepper seed flour (*Capsicum annuum* L.)), which could guide the food industry toward new product development based on the circular bioeconomy.

## 1. Introduction

Due to their sensorial and nutritional value, vegetables are part of nearly every diet all around the world. In recent times, more resources are being used for the purpose of utilizing agricultural by-products into new, highly valuable products or ingredients. Peppers, as favorable vegetables, are botanically classified within the Solanaceae family as a *Capsicum* genus. *Capsicum annuum* L. is a species that is cultivated within the genus [1]. *Capsicum annuum* is a native species that originated from the south of North America, spread to Central America and South America and has a more than 400-year history of cultivation [2]. It has different forms of fruit that vary in size and taste, as well as in names associated with etymology. For instance, in American English, varieties without pungence are called sweet pepper, while those rich in capsaicin (which contribute to a peppery or hot taste) are called hot pepper or chili pepper. In British English, sweet varieties are called peppers and hot varieties chilies, while in Australian English and Indian English, *Capsicum* is the name mostly used for bell peppers (blocky shape) and chili for hotter varieties [3]. 

**Figure 1:**
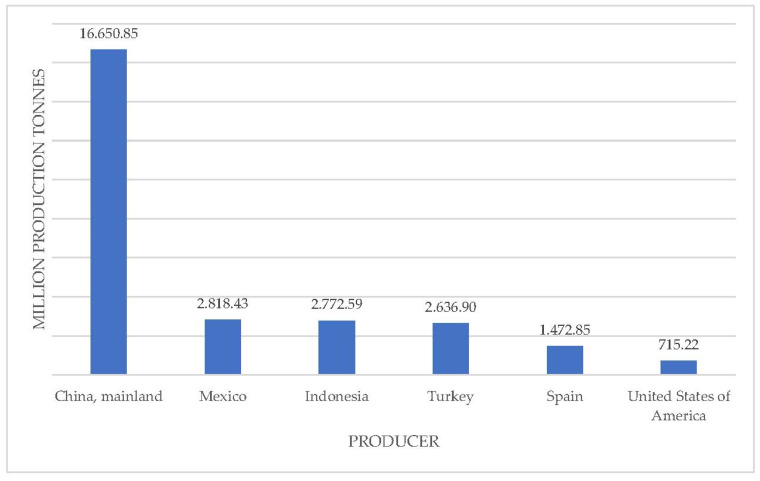
Top producers of chilies and pepper (green) for 2020 [5].

The *Capsicum annuum* L. species is one of the most investigated species under the *Capsicum* genus [4]. Asian producers account for 70% of the global production of *Capsicum annuum* L. (peppers, chili peppers) [5]. The top six chili and pepper producers in the world were China, Mexico, Indonesia, Turkey, Spain and USA, according to annual production in 2020, as shown in Figure 1. The yearly production of pepper shows an increasing trend in recent times [5]; therefore, the effective utilization of larger amounts of pepper processing waste is very important.

**Figure 2:**
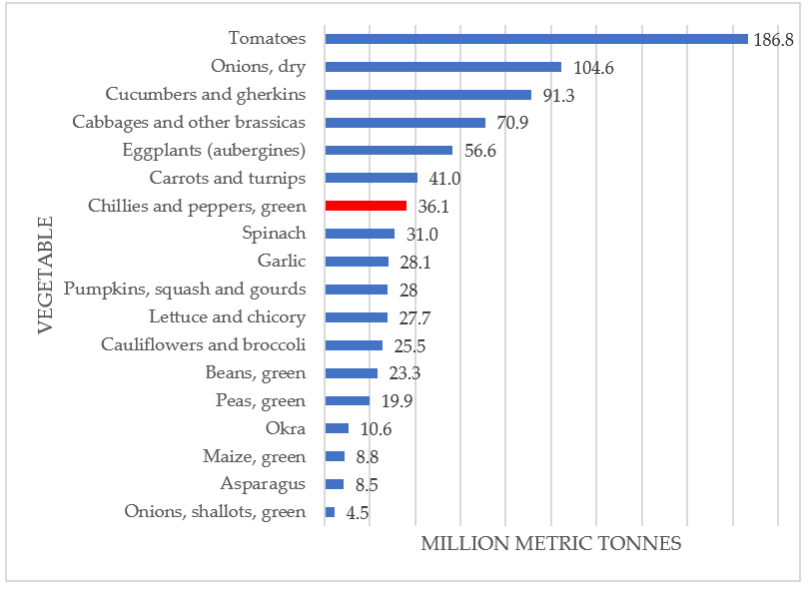
Global production of vegetables (in million metric tonnes) for 2020 [5].

Fresh peppers are a source of vitamin C, provitamin A, carotenoids, phenolic acids and flavonoids. These nutrients are beneficial to human health because of their protective role against certain cancers, in the prevention of gastric ulcers, stimulation of the immune system, prevention of cardiovascular diseases and protection against age-related macular degeneration and cataracts, as documented in the literature [6,7]. Specifically, red peppers have high antioxidant potential due to high total phenolic and flavonoid content [8]. Peppers have a long history of culinary and industrial applications in the world of gastronomy: fresh or baked (salads or side dishes), processed (dehydrated products, pickled products, condiments, sauces, soups, flavoring and coloring purposes). Peppers are among the top 10 vegetables according to annual world production in 2020 (Figure 2).

In a recently published paper [9], pepper seeds, originating from the Croatian pepper varieties *Podravka* and *Slavonka*, were evaluated as new sources of high-value ingredients. Pepper seed oil obtained by cold pressing showed potential for culinary usage, and its nutritional quality can be compared to more expensive vegetable oils on the market.

The aim of this review paper is to reveal the literature data on the quality of two pepper by-products (oil and seed flour). “Pepper seed oil” is a product obtained from pepper seeds by different extraction methods. “Pepper seed flour” is the product obtained from pepper seeds by different grinding methods and may vary in size of the particles.

## 2. Chemical Composition of Pepper Seed Flour and Pepper Seed Oil

### 2.1. Pepper Seeds (Flour)

Pepper seed flour is the product in powder form obtained from pepper seeds by grinding and before the extraction of oil. The chemical composition showed that pepper seeds obtained from the pepper processing industry contain high amounts of dietary fiber, protein and oil [10]. Results on protein, oil, carbohydrate and total dietary content of pepper seeds from several published studies are summarized in Table 1. All seed samples were from the *Capsicum annuum* species, except for those in the study by Jarret et al. [4], who investigated nine *Capsicum* species, between which only two were *Capsicum annuum*, and the study by Firatligil-Durmus and Evranuz [11], who investigated *Capsicum frutescens* L. According to Embaby and Mokhtar [12], pepper seeds contain some antinutritional compounds such as phytic acid, trypsin inhibitors and tannins, so the seeds must be treated to reduce the level of these compounds before incorporating them into food or animal diets.

According to Bostanci et al. [13], pepper seeds (flour) may be used in the development of nutritionally rich and healthy new products such as chocolate spreads. Proteins, dietary fibers and oil in pepper seed flour are found in significant amounts, and the flour can be a valuable ingredient in developing a breakfast sauce formulation prepared with tomato paste and spice mixtures [14]. The main challenge in using pepper seed flour as a functional ingredient is its inherent bitterness, which can be dominant in taste, depending on its dosage in products [14].

#### 2.1.1. Protein Content

Pepper seed proteins have not been extensively researched according to the literature. As per the data shown in (Table 1), pepper seeds provide high amounts of protein. The protein content varied between 13.8% [13] and 28.3% [15]. El-Adaway and Taha [16] reported protein content of 24.4% and also found high levels of lysine, threonine, total aromatic amino acids and tryptophan in pepper seed flour in comparison with data from FAO/WHO [17]. Yilmaz and Hüriyet [18] were the first who report the physicochemical (color, thermal properties, viscosity, molecular weight, amino acid composition, solubility) and functional properties (foaming capacity and stability, water and oil holding capacity, emulsifying activity and stability, least gelling concentration) of pepper seed protein. They extracted protein from grounded defatted pepper seed press cake (meal) using an alkaline extraction–isoelectric point precipitation technique, after which the main physicochemical and functional properties of seed protein were determined. They determined that pretreatment of pepper seeds such as preroasting and enzyme treatment caused loss of seed protein and affected the protein properties. Under the same conditions, the protein yields were 40% for the control sample of pepper seeds, 33% for roasted pepper seeds and 15% for enzyme-treated samples. Furthermore, the authors also concluded that the proteins lost their solubility as a result of seed pretreatment. Hot pepper seeds originating from China’s Northeast showed 21.3% protein content, which represents a good source of proteins [19]. The protein content of red pepper seed before and after roasting was 17.9% and 19.4% according to Gu et al. [20]. The protein content in Croatian varieties of pepper seeds showed 16.5% for the *Slavonka* variety, while the *Podravka* variety showed 16.7% [9]. Multiple factors, such as plant variety, cultivation, climate, ripening stage and the harvesting time of seeds [21], may cause variations in protein and oil yields. Pepper seed contains most of the essential amino acids and can be used as a good protein source for a variety of food applications [13,14]. Firatligil-Durmus and Evranuz [11] established optimum conditions for extracting red pepper seed protein. Maximum yield was obtained when temperature, pH, mixing time and solvent/meal ratio were 31 °C, 8.8, 20 min and 21:1 (*v*/*w*), respectively. Li et al. [22] investigated the functional properties of pepper seed protein isolates and concluded that ultrasound-assisted extraction improved the yield and protein content, as well as some functional characteristics such as oil holding capacity. In the same study, they concluded that pepper seed protein hydrolysates have antioxidant properties to prevent oxidation in food processing. According to in vitro protein digestibility and quality of protein, El-Adaway and Taha [16] found that pepper seed flour had lower digestibility in comparison with watermelon and pumpkin seed flour. It can be connected with the highest tannins content in pepper seed flour. Aw and Swanson et al. [23] found that tannins unfavorably affect the nutritive value of black beans by decreasing the digestibility of proteolytic enzymes. The quality of the protein depends, among others, on the amino acid composition. The most dominant amino acids in pepper seed flour were leucine and sulfur amino acids. Therefore, based on this pepper, seed flour could be used to improve the protein quality of wheat flour that is deficient in lysine. The pepper seed flour, together with watermelon and pumpkin seed flours, absorbed more fat than water, and water absorption capacity was quite high compared with other vegetable proteins such as faba bean flour [24]. Hence, pepper seed flour has the potential to be used in some bakery products, as a meat replacer and as a thickening agent in soups [16].

#### 2.1.2. Carbohydrate and Total Dietary Fiber Content

In the carbohydrate content of pepper seeds, the most dominant component is dietary fiber. Results of carbohydrate and dietary fiber content are shown in Table 1. The data showed 56.3% [12] and 55.1% [11] of total carbohydrates in pepper seeds, which is an abundant amount. Chouaibi et al. [15] found lower carbohydrate content in pepper seeds (43.60%). Furthermore, red pepper seed could be considered a sustainable source of dietary fiber, with levels ranging from 61% [10] to 26% [20], which is significant. The amount of carbohydrates in the *Slavonka* and *Podravka* pepper seed varieties were 3.4 and 3.2%, and the dietary fiber values were 42.1% for *Slavonka* and 41.2% for *Podravka* seed flour [9]. The ratio of insoluble and soluble dietary fiber was around 10:1, according to Azabou et al. [10]. High content of insoluble fiber in pepper seeds could potentially represent a new ingredient in the food industry, enhancing indigestible insoluble compound levels [25]. 

Pepper seed flour had a higher value of crude fiber content (34%) than watermelon and pumpkin seed kernels (4.9%; 4.4%), according to El-Adaway and Taha [16]. They assumed that, according to the obtained content, pepper seed could be a good source of dietary fiber. Zou et al. [19] found 38.8% of total dietary fiber content in hot pepper seeds. The high amount of fiber in pepper seeds could benefit human health because of its capacity in preventing obesity, cardiovascular problems, diabetes and colon cancer [26].

The latest research showed that intake values for dietary fiber were 16.7 g/day for men and 15.6 g/day for women, which is below the recommended levels (adult women: 28 g/day; adult men: 36 g/day) [27]. Therefore, fortification with dietary fiber in the food industry and developing new fiber-rich products is becoming increasingly important [28]. According to the EU regulation [29], there should be at least 3% of dietary fiber in a certain product for it to be considered “a source of fiber” and 6% of dietary fiber to be “rich in fiber”. Dordevic et al. [30] stated that sensory properties can be significantly affected by the addition of more than 1% of bamboo fiber in fruit jams. The same situation could be in the fortification of the product with pepper seed flour because of its bitterness [14]. Since this study showed some negative effects of using pepper seed flour as a source of dietary fiber in products, this aspect needs further investigation.

Since this study showed some negative effects of using paprika seed flour as a source of fiber in products, this aspect needs to be further investigated. 

These investigations should be about pepper seed flour dosation in different kind of products. The question is in what dasage the pepper seed flour cause bitternes of the specific product. 

**Table 1:** Protein, oil, carbohydrate and dietary fiber content (%) of pepper seeds.

Sample Type	Total Dietary Fiber	Carbohydrate	Protein	Oil	References
*Capsicum annuum* L. (*Podravka* variety)	41.2	3.2	16.7	27.2	[9]
*Capsicum annuum* L. (*Slavonka* variety)	42.1	3.4	16.5	26.7	[9]
*Capsicum annuum* L.	34.9	/	24.4	25.6	[16]
*Capsicum annuum* L. (10 varieties from Turkey and Italy)	/	/	/	8.5–32.6	[31]
*Capsicum frutescens* L.	/	55.1	26.0	/	[11]
*Capsicum annuum* L.	/	56.3	19.3	19.6	[12]
*Capsicum annuum* var. *annuum*, var. *glabriusculum Capsicum* (*boccatum*, *chinense*, *frutescens*, *pubescens*)	/	/	/	21.1–28.1	[4]
	/	/	/	18.3–24.4	
*Capsicum annuum* (capia variety)	/	/	21.5	13.6	[1]
*Capsicum annuum* (cv. “Jinta”; hot pepper cultivar)	38.8	/	21.3	23.7	[19]
*Capsicum annuum* L.	26.0	/	17.9	16.0	[20]
*Capsicum annuum* L.	61.0	/	18.3	11.0	[10]
*Capsicum annuum* (capia variety)	/	/	13.8	21.6	[13]
*Capsicum annuum* L. (varieties—SZ–20, meteorite, sun dried; growing seasons 2013 and 2014)	/	/	/	9.0–12.0	[32]
*Capsicum annuum* L. (red pepper)	/	43.6	28.3	18.4	[15]
*Capsicum annuum* L.	/	/	/	16.7	[33]

(/) Not investigated in the study.

### 2.2. Pepper Seed Oil

Published papers have determined that pepper seeds are an excellent source of high-quality oil in comparison with edible oils such as peanut oil [34] and sunflower oil [12]. The oil has an orange-red color, a pleasant odor and the characteristic taste of pepper [9] The present review provides data on the nutritional diversity of pepper seed oil in the *Capsicum* genus.

According to the *Capsicum annuum* pepper seed oil content in Table 1, El-Adaway and Taha [16] found that seeds had an oil content of 25.6%, while Azabou et al. [10] found lower values of oil (11%), similar to the study of Konscek et al. (9–12%) [32]. Matthäus and Őzcan [31] investigated 10 samples of *Capsicum annuum* from Turkey and Italy, and oil content varied from 8.5% to 32.6%. Seed oil content varied due to species and plant varieties of the pepper [4]. El-Adaway and Taha [16] evaluated the chemical composition of pepper seed oil for the first time. They reported that *Capsicum annuum* seeds are a good source of protein (24%), oil (26%) and fiber (35%). Hot pepper seeds showed to be a good source of oil (23.7%), according to Zou et al. [19]. 

Reports in the scientific literature on pepper seed oil content and the fatty acid composition of oil have mainly been limited to oil obtained from *Capsicum annuum* seeds. Jarret et al. [4] showed variations from 18.3% to 24.4% in seed oil content between five cultivated species of the *Capsicum* genus (*C. baccatum* var. *pendulum*, *C. baccatum* var. *baccatum*, *C. chinese*, *C. frutescens*, *C. pubescens*). Furthermore, these results showed variations in seed oil content from 21.1% to 28.1%, even between two varieties of *Capsicum annuum* seeds (*C. annuum* var. *annuum*, *C. annuum* var. *glabriusculum*). Results in the same study showed minimal environmental effects on seed oil content, which included different regions and growing seasons. The average difference in seed oil content between different regions and growing seasons was only 2.4%. Jarret et al. [4] showed a significant correlation between the pepper seed weight and seed oil content of the cultivated species. The same variations between seed oil content were found in the four wild species of *Capsicum* (10.8–21.2%), according to Jarret et al. [4]. 

Embaby and Mokhtar [12] found higher oil content in sweet pepper seeds (19.6%) than in lantana seeds (11.0%) but lower than in nabak seed kernels (30.2%). The capia pepper seed variety *Capsicum annuum* L. from the processing seasons 2012 and 2016 was investigated by Yilmaz et al. [1] and Bostanci et al. [13]. Seed oil content was 13.6% in the 2012 season and 21.6% in the 2016 season. It is evident that season had a great influence on oil content, which is not in agreement with Jarret et al. [4]. Seed oil content was similar according to other authors, namely Gu et al. [20] (16%), Chouaibi et al. [15] (18.4%) and Ma et al. [33] (16.7%). Reports from Cvetković et al. [9] showed that oil content was similar in both seed varieties, from 26.7% for the *Slavonka* variety to 27.2% for the *Podravka* pepper seed variety. 

#### 2.2.1. Oil Production

**Figure 3:**
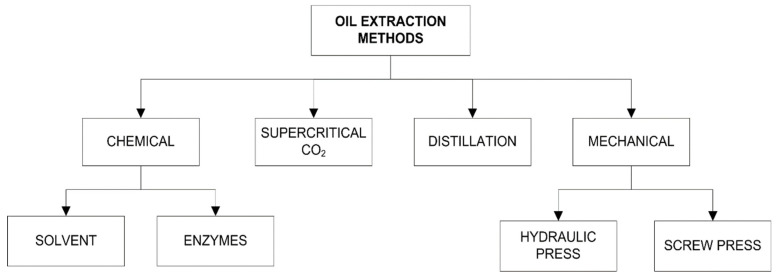
Oil extraction methods [36].

According to all the data given above, oil content is influenced by various factors such as pepper variety, region and growing season, the process used for obtaining oil is also important. Pepper seed oil can be obtained by various methods such as solvent extraction, mechanical pressing and supercritical CO_2_ extraction. The applied method has a direct impact on the quality and quantity of the obtained oil. The two most important processes for oil production are physical and chemical (Figure 3), of which the most important physical processes are discontinuous pressing with a hydraulic press and continuous pressing with a screw press. Applying heat treatment before or during pressing generally improves oil efficiency, but this can negatively affect oil quality by reducing oxidative stability parameters. When producing cold-pressed oil, attention should be paid to the temperature of the crude oil, which should not exceed 50 °C. For this purpose, pressing must be performed in milder conditions and at lower pressure. Due to these facts, the amount of residual oil in cold pressing is significantly higher than in hot pressing. Cold-pressed oil has a more intense taste, odor and color, which makes this type of oil more expensive than oil obtained by hot pressing. Although oils obtained by mechanical pressing are of high quality, they cannot be completely extracted by this method, so a lot of oil remains in the cake [35].

The method of chemical extraction is based on the use of enzymes or solvents to extract oil from the raw material. In enzyme treatment, the selection of the appropriate enzyme is very important to improve oil yield. The solvent extraction method uses hexane (Soxhlet extraction) as the solvent and can yield up to 99% oil, but the oil quality is lower, and the organic solvent remaining in the oil is undesirable and has a certain toxicity, which is a big problem. A refining process is required to remove the organic solvent and to obtain edible oil. After oil extraction, defatted cake is a valuable source of protein for animal feed. Organic solvents also extract some nontriglycerides, which do not appear in oil obtained by pressing. Furthermore, the biggest problem is the presence of volatile organic impurities in the final product, which can affect the quality of the oil [35].

More recently, supercritical CO_2_ extraction has been increasingly used as a process to extract oil from seeds. It is still a relatively new method in the extraction of edible oils mainly due to the very high investment costs of the equipment and the high energy demand. However, today, “green” products and technologies are needed to replace conventional types. With the introduction of new green extraction techniques such as supercritical CO_2_ extraction, conventional methods are becoming decreasingly popular. The reasons may be toxic chemicals used in the solvent extraction process and oil yield that is not achieved completely by cold pressing, and the quality of the oil is significantly higher than that obtained by solvent extraction [35,36].

In terms of extraction methods, Chouaibi et al. [15] investigated the chemical and antioxidant potential of pepper seed oil obtained by different extraction methods (Soxhlet, cold pressing, supercritical CO_2_ and microwave-assisted extraction process). The highest and lowest contents of linoleic acid were found in microwave-assisted (76.5%) and Soxhlet (73.7%)-extracted pepper seed oils, and high content in total tocopherols was found in microwave-extracted red pepper seed oil (136.5 mg/kg). They concluded that the peroxide value of Soxhlet-extracted oil was significantly higher than those of other oils obtained by different extraction methods, and there were no big differences between cold pressing (5.4 meq O_2_/kg oil) and supercritical CO_2_ (5.2 meq O_2_/kg oil)-extracted pepper seed oil in terms of peroxide value. A high peroxide value in pepper seed oil, as in vegetable oil in general, shows overheating and inappropriate storage conditions, and it is the most common parameter that indicates oil oxidation. The results of oxidative stability showed the highest value in microwave-obtained oil and the lowest value in Soxhlet-obtained oil. It could be related to phenolic content, but reduced oil stability could be because of more peroxides resulting from prolonged exposure of the oil to high temperatures during extraction, resulting from prolonged exposure of the oil to temperature.

The results are in accordance with Yilmaz et al. [1] for the peroxide value in pepper seed oil obtained by cold pressing, which was 4.6 to 5.8 meq O_2_/kg oil. According to Ma et al. [33], pepper seed oil, obtained by solvent extraction, had the highest acid value (4.0 mg KOH/g) and peroxide value (1.8 mmol/kg) due to the prolonged extraction time in the solvent extraction process that sped up the process of hydrolysis and oil oxidation. After they compared the quality of cold-pressed pepper seed oil, ultrasound-extracted pepper seed oil and solvent-extracted seed oil, they concluded that the pressure-assisted extraction method was the most appropriate method for extraction of high-quality oil, and the highest oil extraction efficiency (83%) was reached with the following parameters: 370 MPa (pressure), 50 °C (extraction temperature) and 5.7 min (extraction time). 

Optimum conditions (extraction pressure, extraction temperature and added concentration of modifier), in terms of supercritical fluid extraction, were investigated by Li et al. [37]. The same study showed that ethanol as a modifier in supercritical fluid extraction can affect oil yield, as well as on free fatty acid (FFA) content. Under the same extraction conditions, total FFA without ethanol was 939.9 µg mL^−1^ and with ethanol was 1179 µg mL^−1^. According to these results, ethanol had negative effects on oil quality since FFA content is one of the most frequently used parameters for determination of oil quality. 

Except peroxide value, acid value (AV) can be used to measure oil quality too in terms of stability and it is positively correlated with free fatty acid content (FFA). Considering recommended AV for cold-pressed and virgin oil, AV in cold-pressed pepper seed oil of 3 mg KOH/g Oil [16] and 1.4 mg KOH/g Oil [4] was acceptable. 

According to our last paper published on this topic [9], pepper seed oil obtained by cold pressing had better results according to nutritional quality, sensory evaluation and consumer testing compared with pepper seed oil obtained by supercritical CO_2_ extraction. The pepper seed oil obtained by cold pressing had a more intense color, odor and aroma, as well as pleasant spiciness, which contributed to greater acceptance in the consumer test between 60 consumers. 

Additionally, after oil extraction of a defatted meal rich in protein, fiber and carbohydrates remained, and as such, it can be a valuable raw material in the production of new functional and nutritionally enriched foods [38]. 

#### 2.2.2. Fatty Acid Profile of Pepper Seed Oil

The predominant fatty acid in pepper seed oil is linoleic acid. Saturated fatty acids are palmitic and stearic acids, and oleic and linoleic acids are the main unsaturated fatty acids. According to Table 2, linoleic acid content ranged from 67.8% [16] to 77.9% [4], oleic acid content was between 4.6% [39] and 14.6% [16], palmitic acid content was between 10.6% [4] and 14.4% [16] and stearic acid content was between 2.4% (Chouaibi) and 4.1% Matthaus). Konscek et al. [32] showed that different varieties and growing seasons did not change the fatty acid composition of pepper seed oil. Differences in fatty acid composition between species and varieties were not significant. The predominant fatty acid in *Capsicum annuum* seed oils was linoleic acid, ranging from 69.5% to 74.7% in different varieties [31] and from 70.9% to 74.3%, according to Koncsek et al. [32]. The study [9] showed that linoleic acid was dominant in the oils produced from *Podravka* and *Slavonka* seeds. However, the cold-pressed oil of the *Slavonka* variety had the highest amount of C18:2 (77.7%), while the SC-CO_2_-extracted oil had the lowest amount (74%), so it is very important to investigate oil characteristics in terms of antioxidant potential and dominant bioactive compounds as well. 

Therefore, the results from Table 2 show limited variations in the amount of certain fatty acids C18:2 (67.8–77.9%), C18:1 (4.6–14.6%), C18:0 (2.4–4.1%) and C16:0 (10.6–14.4%). Matthaus et al. [31] showed a similar conclusion, where the variation in linoleic acid (C18:2) between *Capsicum annuum* varieties was small (69.5–74.7%), as well as the other determined fatty acids (C16:0, 10.7–14.2%; C18:0, 2.5–4.1%; C18:1, 8.9–12.5%). Azabou et al. [10] determined that among the polyunsaturated fatty acids (PUFAs), C18:2 (70.9 ± 0.8) was dominant, C18:1 (12.2 ± 0.2%) was the dominant monounsaturated fatty acid (MUFA), while C16:0 and C18:0 were the dominant saturated fatty acids (SFA) (11.9 ± 0.3 and 3.54 ± 0.2).

Due to the high levels of linoleic acid (C18:2, 71.6%) in sweet pepper seed oil, Embaby and Mokhtar [12] suggested its usage as edible cooking or salad oil. These findings correlate with the research by Jarret et al. [4], which showed a general similarity in the fatty acid composition of species within a genus. Additionally, PUFAs present a health benefit because they reduce total cholesterol and body fat [10]. According to Regulation No. 1924/2006 [29] and Commission Regulation EU No. 432/2012 [40], two kinds of statements can be used for oils: Linoleic acid contributes to the maintenance of normal blood cholesterol levels. The beneficial effect is obtained with a daily intake of 10 g of linoleic acid and replacing saturated fats with unsaturated fats in the diet contributes to the maintenance of normal blood cholesterol levels (MUFA and PUFA are unsaturated fats). Konscek et al. [32] showed in their study that 10 g of pepper seed oil as salad oil can ensure 7.0–7.4 g linoleic acid, which covers the 70–74% suggested beneficial minimum daily intake.

**Table 2:** Fatty acid composition (%) of pepper seed oil (*Capsicum annuum*).

Sample Type	Oil Extraction Method	Palmitic C16:0 (%)	Stearic C18:0 (%)	Oleic C18:1 (%)	Linoleic C18:2 (%)	References
*Capsicum annuum* L. (*Slavonka* variety oil)	SC-CO_2_ extraction	11.0	3.0	8.4	74.0	[9]
*Capsicum annuum* L. (*Slavonka* variety oil)	Cold pressing	10.9	3.0	8.4	77.7	[9]
*Capsicum annuum* L. (*Podravka* variety oil)	SC-CO_2_ extraction	11.9	3.3	8.8	76.0	[9]
*Capsicum annuum* L. (*Podravka* variety oil)	Cold pressing	10.8	3.4	10.4	75.4	[9]
*Capsicum annuum* L.	Petroleum ether extraction	13.8	3.7	14.6	67.8	[16]
*Capsicum annuum* L. (10 varieties from Turkey and Italy)	Petroleum ether extraction	10.7–14.2	2.5–4.1	8.9–12.5	69.5–74.7	[31]
*Capsicum annuum* L.	Petroleum ether extraction	12.3	3.2	13.0	71.6	[12]
*Capsicum* (7 cultivated species)	Heptane extraction oil	10.6–14.4	2.7–4.0	5.4–7.6	73.9–77.9	[4]
*Capsicum annuum* (capia variety)	Cold pressing	11.6	4.0	11.10	71.1	[1]
*Capsicum annuum* L.	Cold pressing subcritical butane extraction	11.5–11.6 11.4–11.7	2.7–2.9 2.5–3.0	9.8–9.9 10.0–9.6	72.7–72.4 73.0–72.1	[20]
*Capsicum annuum* L.	Hexane extraction	11.9	3.5	12.2	70.9	[10]
*Capsicum annuum* L. (varieties—SZ–20, Meteorit, growing seasons 2013 and 2014)	Cold pressing	11.1–12.2	3.1–3.8	7.9–9.6	70.8–74.3	[32]
*Capsicum annuum* L. ssp. *macrocarpum* (Strumica valley)	Cold pressing	10.8	/	4.6	69.6	[39]
*Capsicum annuum* L. (red pepper)	Cold pressing, hexane extraction, supercritical CO_2_, microwave-assisted extraction	13.4 12.3 11.2 11.4	2.5 2.5 2.4 2.4	9.2 9.8 8.8 8.2	73.7 73.9 76.3 76.5	[15]
*Capsicum annuum* L.	Supercritical propane extraction	11.6	2.4	11.6	72.6	[41]
*Capsicum annuum* L.	Hexane extraction, ultrasound-assisted, pressure-assisted extraction	13.4 13.4 13.3	2.8 2.8 2.7	9.2 9.0 9.1	72.4 72.5 72.6	[33]

(/) Not investigated in the study.

In comparison with sunflower, soybean or peanut oil, pepper seed oil has higher amounts of palmitic and linoleic acid [42,43,44] and is classified as an edible oil rich in linoleic acid. 

There were no significant differences among fatty acid content between unroasted and roasted pepper seed oil, as well as among mechanical extraction and subcritical butane extraction [20]. Zhang et al. [41] extracted pepper seed oil by subcritical propane extraction and showed that extraction time had no effect on fatty acid composition. 

Veličkovska et al. [39] showed that linoleic acid (69.6%) was the most dominant fatty acid in paprika seed oil. Yilmaz et al. [1] applied two different pretreatments on pepper seeds (preroasting and enzyme treatment) before cold pressing. Their results showed no big differences in fatty acid composition and content of oil samples obtained by preroasted and enzyme treatment seeds against the control group (no pretreatment). They found four dominant fatty acids in pepper seed oil: palmitic acid (16.0%), stearic acid (4.0%), oleic acid (11.1%) and linoleic acid (71.1%), the most dominant. 

Ma et al. [33] compared the chemical composition and characteristics of pepper (*Capsicum annuum* L.) seed oil obtained by pressure-assisted, ultrasound-assisted and conventional solvent extraction (*n*-hexane as a solvent) and concluded that extraction methods had no influence on fatty acid composition. Chouaibi et al. [15] compared pepper seed oil obtained by Soxhlet, cold pressing, supercritical CO_2_ and microwave-assisted extraction.

#### 2.2.3. Total Phenolic, Flavonoid and γ-Tocopherol Content

Polyphenols, tocopherols, phytosterols are important bioactive compounds found in pepper seed oil [1]. The extraction technique greatly influences total phenolic compounds and the γ-tocopherol content of pepper seed oils, and total phenolic compounds affect antioxidant activities [15]. Cvetković et al. [9] showed high differences between γ-tocopherol content in oils obtained by different extraction methods. In the *Podravka* variety, the content of γ-tocopherol was 80.1 mg/100 g (cold pressing) and 65.3 mg/100 g (supercritical CO_2_ extraction) and in the *Slavonka* variety, 65.3 mg/100 g (cold pressing) and 16.0 mg/100 g (supercritical CO_2_ extraction).

There were different results for γ-tocopherol, total phenolic and flavonoid content in pepper seed oil among different extraction methods, according to the data given in Table 3. Tocopherols are natural lipid antioxidants that can scavenge free radicals in photosynthetic tissues and inhibit the peroxidation of oils [45,46]. Hence, previous studies have shown the most dominant was γ-tocopherol among analyzed tocopherols. Data for γ-tocopherol, the most dominant tocopherol in pepper seed oil, are given in Table 3. A recent study suggests that γ-tocopherol may be superior to α-tocopherol in preventing the oxidation of low-density lipoproteins and delaying thrombus formation [47]. Jiang et al. [48] reported that the plasma concentrations of γ-tocopherol are inversely associated with the incidence of cardiovascular diseases and prostate cancer. 

γ-Tocopherol was the main tocopherol, followed by α-tocopherol, δ-tocopherol and β-tocotrienol, according to Matthaus and Őzcan [31]. The amount of γ-tocopherol varied from 306.6 mg/kg (Italy) to 602.6 mg/kg (Turkey). Additionally, bitter pepper seeds have higher content of vitamin E compared to those with a sweet taste. Koncsek et al. [32] did not detect β-tocopherol and δ-tocopherol in cold-pressed spice pepper seeds oil. The amounts of α-tocopherol (13.5–16.4 mg/100 g) were significantly lower compared to the dominant γ-tocopherol (57.9–83.6 mg/100 g). A large variation in the γ-tocopherol content of pepper seed oil can be affected by the growing season, as well as by the used extraction methods [15]. Results for γ-tocopherol were: 113.2 mg/kg (cold pressed) and 94.4.mg/kg (hexane); 130.6 mg/kg (supercritical CO_2_ extraction) and 136.5 mg/kg (microwave-assisted extraction). The data listed in Table 3 report that cold-pressed seed oil had higher γ-tocopherol content (80.4 mg/kg) than seed oil obtained by conventional solvent extraction (Soxhlet) (77.1 mg/100 g), according to Ma et al. [33]. Yilmaz et al. [1] found different contents of γ-tocopherol in control (164.4 mg/100 g), preroasted (152.9 mg/100 g) and enzyme (169.1 mg/100 g)-treated seed oil. Roasted samples showed a lower content of γ-tocopherol than other samples. Zhang et al. [41] found a γ-tocopherol content (21.9 mg/100 g oil) of pepper seed oil obtained by subcritical propane extraction in the first stage of extraction. Gunstone [42] showed that cold-pressed spice pepper oils can be protected from autoxidation (γ-tocopherol provided oxidative stability). Zhang et al. [41] confirmed these findings and showed that capsaicins and tocopherols protected pepper oil from thermal oxidation during frying.

There are only a few available studies about total phenolic and flavonoids either in oil or in pepper seeds shown in Table 3. The phytochemicals in pepper seeds and oil became interesting after the annual increase in red pepper production. Natural antioxidants have become increasingly popular because of the toxic anticarcinogenic outcomes of synthetic antioxidants [49,50]. Therefore, sources of natural antioxidants such as pepper processing waste are increasingly being explored, as well as polyphenols as valuable phytochemicals in such materials. 

Phytochemicals such as polyphenols and flavonoids affect the higher antioxidant activity and prevent the risk of degenerative diseases [51,52].

Howard et al. [53] reported that total phenolic and flavonoid concentrations depend on the pepper cultivar. Peterson and Dwyer [54] proposed a botanical classification scale for flavonoid concentration, which rates foods as low (0.1–39.9 mg/kg), moderate (40–99.9 mg/kg) and high (>100 mg/kg).

Sim and Sil [8] found that the total phenolic content of red pepper seed extract was lower (29.1 mg gallic acid equivalent GAE/g) than the phenolic content of red pepper pericarp (47.5 mg GAE/g). Red pepper pericarp extracts had higher scavenging activity than red pepper seed extract at the same concentration. Therefore, red pepper pericarp was a significantly stronger scavenger for DPPH (2,2-diphenyl-1-picryl-hydrazyl-hydrate) radicals. The results of the (DPPH method) radical assay indicate the strong association between the antioxidant activity and level of phenolic compounds.

The total amount of phenolic compounds correlates with antioxidant activity according to previous studies [55]. In terms of total flavonoid content, red pepper pericarp was found to be higher in flavonoids (27.5 mg catechin equivalent CAE/g) compared with red pepper seed (21.3 mg CAE/g) [8]. Amarowicz et al. [56] reported that tannins from canola and rapeseed had a positive effect on scavenging efficiency. Considering that, there was a connection between tannins in pepper seeds and their antioxidant activity. Pepper seed oil had the highest quantity of total phenolic content (117.4 mg gallic acid/L oil) after sesame seed oil (214.1 mg gallic acid/L oil), according to Veličkovska et al. [39]. In correlation, pepper seed oil had the highest antioxidant potential according to the Trolox equivalent antioxidant capacity (TEAC) method (97.9 Trolox/L oil) and β-carotene assay (48.6% inhibition of linolenic acid oxidation). This can be related to the fact that pepper seed oil is a rich source of carotenoids [57].

According to Chouaibi et al. [15], total phenolic compounds ranged from 8.3 to 12.6 mg/100 g oil. Total flavonoid content ranged from 1.6 to 2.1 mg/100 g oil. Pepper seed oil obtained by supercritical CO_2_ extraction contained a huge quantity of total polyphenols, followed, in decreasing order, by microwave, Soxhlet and cold pressing. The most significant phenolic acid in pepper seed oil is gallic acid, and flavonoid is routine. The pepper seed oil obtained by SC-CO_2_ had the highest gallic acid (6 mg/100 g) and routine content (1.6 mg/100 g) in comparison with oil obtained by microwave, Soxhlet and cold pressing. This suggests that the content and type of polyphenolic substances in pepper seed oil are variable and mainly depend on the used extraction methods.

According to antioxidant activity, the highest DPPH value was found in oil obtained by solvent extraction (hexane, 5.4 mg/100 mL) and the lowest in oil obtained by microwave-assisted extraction (0.8 mg/100 mL). Regarding ABTS radical scavenging activity (2,2-azinobis-(3-ethylbenzothiazoline-6-sulfonic acid), it was the lowest in oil obtained by microwave-assisted extraction (0.2 mg/mL) and the highest in oil obtained by solvent extraction (4.5 mg/mL). This indicates that oil obtained by solvent extraction has the lowest antioxidant activity, and microwave-assisted oil has the highest antioxidant activity, which partly correlates with the study of Ma et al. [33], where it was concluded that solvent extraction oil had the lowest antioxidant capacity, while cold-pressed oil had the highest antioxidant capacity.

Total phenolic content was the highest in the control sample (24 µg gallic acid GA/100 g) and the lowest in oil obtained from preroasted pepper seeds (18.3 µg GA/100 g), which indicated that roasted and enzyme treatment affected total phenolic content [1]. Antioxidant capacity measured by TEAC showed a correlation between total phenolic content and antioxidant capacity, which indicated that after roasting and enzyme treatment, there was a significant reduction in antioxidant capacity. 

A correlation between antioxidant capacity and total phenolic content was found in the study by Cvetković et al. [9]. Polyphenol content was 158.2 mg/100 g in the *Podravka* variety of pepper seeds and 149.9 mg/100 g in the *Slavonksa* variety of pepper seeds. Antioxidant capacity was measured by antioxidant power (AP) [58]. Antioxidant power for *Podravka* pepper seed flour was 107 AU and 70 AU for *Slavonka*, which is in accordance with the polyphenol content found in these two samples. The highest content of γ-tocopherol was found in *Podravka* pepper seed oil obtained by cold pressing (80.1 mg/100 g), and it is also in correlation with AP in *Podravka* pepper seeds (107 AU).

Phytosterols are a group of minor components with a structure similar to cholesterols. They are an important nutritional compound that can reduce serum LDL cholesterol, as well as atherosclerotic risk. The highest level of total phytosterols was observed in pepper seed oil (over 5500 mg/kg) in the study by Veličkovska et al. [39]. The most dominant phytosterol in pepper seed oil was Δ5-avenasterol with the level of 1141 ± 8.1 mg/kg, which amounts to one-fifth of the total phytosterols content in pepper seed oil. 

A similar level of total phytosterols was found in pepper seed oil of the Turkish variety (6643.5 ± 19.9 mg/kg) [31]. Silva et al. [59] found three phytosterols, campesterol, stigmasterol and β-sitosterol, with the domination of campesterol and β-sitosterol in pepper seed oil. These results showed that pepper seed oil is a valuable source of sterols and can act as an antioxidant and as an antipolymerization agent in the frying process. Therefore, the determination of the smoke point in pepper seed oil will be an interesting parameter for further investigations.

## 3. Product Development: Valorization of Pepper Seeds

Pepper seeds are usual by-products of the processing of pepper fruits into other products. Due to their proven nutritional and functional characteristics, paprika seeds potentially represent a new valuable raw material either for oil production or protein and fiber extraction. Pepper seed oil can be obtained by several extraction methods. After the cold-pressing method, high-value protein and fiber can be obtained from leftover defatted pepper seed meal (press cake) [13]. 

Pepper seeds can be ground after drying, and the obtained flour can be utilized in new product development. Based on its chemical and sensorial characteristics, pepper seed flour can be applied as an ingredient in new product development to create products with added value [60]. This study showed that pepper seeds can be utilized in minimally processed innovative food products.

Table 4 shows two studies as an example of using product development in the valorization of by-products. In the study of Bostanci et al. [13], capia pepper seeds (*Capsicum annuum* capia variety) were valorized through the new product development of spreadable pastes, based on the addition of a certain amount of capia pepper seed flour in recipes. The new spreadable pastes were rich in linoleic acid, sterols, tocopherols and dietary fibers in comparison to commercially available similar products. According to Yilmaz [14], pepper seed flour has a characteristic peppery flavor and a certain level of bitterness due to its composition. However, due to its overall acceptable sensory characteristics, pepper seed flour in the amount of 20% was successfully used in the development of vegetable and spicy sauces. 

In this review, the data showed that pepper seed oil and pepper seed flour have promising applications in new product development. However, further investigations are necessary in terms of the dosage of pepper seed oil and pepper seed flour in different types of products (soups, pastes, meals, sauces, spice oils) for the best sensory properties of the finished product. To the best of our knowledge, there are no literature data connected with the application of pepper seed cake (a by-product of pressing pepper seeds) in food products. However, due to its nutritive value, the authors believe that pepper seed cake could be interesting for further application and investigation. 

## 4. Conclusions

The literature review of the quality of pepper seed by-products in this paper showed the significant influence of different extraction methods applied among *Capsicum* species and *Capsicum annuum* varieties. In pepper seed oil, protein and total dietary fiber content significantly varied among *Capsicum* species and *Capsicum annuum* varieties, while fatty acid composition did not. On the other hand, total phenolic, flavonoid and γ-tocopherol content were significantly influenced by the applied extraction methods. Similarly, pepper seed flour had high levels of protein and dietary fiber, fatty acids and carbohydrates. Consequently, pepper seed oil and flour could be considered highly valuable products and/or ingredients in the development of new and sustainable products. Culinary application of pepper seed oil has promising potential in gastronomy worldwide due to its well-accepted sensory attributes. However, its usage is likely to be related to consumers′ openness and cultural backgrounds. 

## Figures and Tables

**Table 3:** Total phenolic, flavonoid and tocopherol contents of pepper seed, pericarp and pepper seed oil obtained by different extraction methods.

Sample Type	Oil Extraction Method	γ-Tocopherol	Total Phenolic	Flavonoids	References
*Capsicum annuum* L. oil (*Slavonka* variety)	SC-CO_2_ extraction	16.0 mg/100 g	/	/	[9]
*Capsicum annuum* L. oil (*Slavonka* variety)	Cold pressed	65.3 mg/100 g	/	/	[9]
*Capsicum annuum* L. oil (*Podravka* variety)	SC-CO_2_ extraction	44.7 mg/100 g	/	/	[9]
*Capsicum annuum* L. oil (*Podravka* variety)	Cold pressed	80.1 mg/100 g	/	/	[9]
*Capsicum annuum* L. (*Slavonka* variety) pepper seeds	/	/	149.9 mg/100 g	/	[9]
*Capsicum annuum* L. (*Podravka* variety) pepper seeds	/	/	158.2 mg/100 g	/	[9]
*Capsicum annuum* L. red pepper seed	/	/	29.1 mg GAE/g	21.3 mg CAE/g	[8]
*Capsicum annuum* L. red pepper pericarp	/	/	47.5 mg GAE/g	27.5 mg CAE/g	[8]
*Capsicum annuum* L. (10 varieties from Turkey and Italy) oil	Petroleum ether	306.6–602.6 mg/kg	/	/	[31]
*Capsicum annuum* L. oil (capia variety)	Cold pressed from roasted and enzyme-treated seeds	152.9–169.1 mg/kg	18.3–24.0 µg GA/100 g	/	[1]
*Capsicum annuum* L. seeds	/	/	21.5 mg GAE/g	43.4 µg QE/g	[10]
*Capsicum annuum* L. oil (varieties—SZ-20, meteorite, sun dried; growing seasons 2013 and 2014)	Cold pressed	56.9–83.6 mg/100 g	/	/	[32]
*Capsicum annuum* L. ssp. *macrocarpum* oil (Strumica valley)	Cold pressed	25.7 mg/100 g	117.4 mg GAE/L oil	/	[39]
*Capsicum annuum* L. oil	Subcritical propane extraction	21.9 mg/100 g	/	/	[41]
*Capsicum annuum* L. oil	Cold pressed	113.2 mg/kg	8.3 mg/100 g	1.6 mg/100 g	[15]
	Hexane extraction	94.4 mg/kg	10.5 mg/100 g	1.8 mg/100 g	
	Subcritical CO_2_	130.6 mg/kg	12.6 mg/100 g	2.1 mg/100 g	
	Microwave-assisted extraction	136.5 mg/kg	11.2 mg/100 g	1.9 mg/100 g	
*Capsicum annuum* L. oil	Cold pressed	80.4 mg/kg	/	/	[33]
	Hexane extraction	77.1 mg/kg	/	/	
	Ultrasonic assisted	74.5 mg/kg	/	/	

(/) Not investigated in the study.

**Table 4:** By-product valorization through product development.

Ingredient	Dosage (%)	New Product Development	Conclusion	References
Pepper seed flour	20.0	Breakfast sauce (vegetable and spice type)	Pepper seed flour could be valorized in various types of breakfast sauce, but bitterness should be moderate during such applications	[14]
Pepper seed flour	23.7–30.1	Spreadable pastes (chocolate and molasses type)	Valorization of pepper seeds flour in spreadable product formulations is possible, as well as creation nutritive valuable products	[13]

## Data Availability

Not applicable.
